# Effect of soil moisture on soil disintegration characteristics of different weathering profiles of collapsing gully in the hilly granitic region, South China

**DOI:** 10.1371/journal.pone.0209427

**Published:** 2018-12-31

**Authors:** Dong Xia, Bingqin Zhao, Daxiang Liu, Yusong Deng, Hu Cheng, Yujie Yan, Shuwen Ding, Chongfa Cai

**Affiliations:** 1 Engineering Research Center of Eco-environment in Three Gorges Reservoir Region, Ministry of Education, College of hydraulic and environmental engineering, China Three Gorges University, Yichang, China; 2 College of Civil Engineering and Architecture, China Three Gorges University, Yichang, China; 3 College of Resources and Environment, Huazhong Agricultural University, Wuhan, China; RMIT University, AUSTRALIA

## Abstract

Collapsing gully erosion is the main important and specific soil erosion type in the red soil region of tropical and subtropical South China. Knowledge of the soil disintegration characteristics within different weathering profiles (surface layer, red soil layer, sandy soil layer and detritus layer) and its relationships with soil particle size distribution and soil properties is important in understanding the mechanism of the forming process and development of the collapsing gully. In this paper, we conducted an experiment on four collapsing gullies located four counties (Tongcheng County, Gan County, Anxi County and Wuhua County) in the hilly granitic region of southern China. The anti-disintegration ability of the different weathering profiles with two different moisture conditions (the air-dried condition and the natural state condition) were determined by the anti-disintegration index (*Kc*) and measured by the submerging test. The results show that the coarse particles are higher in the sandy soil layer and the detritus layer of collapsing gully than that in the surface layer and the red soil layer, but the finer particles show the inversed order. The *Kc* values reduce significantly from the surface layer to the detritus layer. In the surface layer and the red soil layer, the *Kc* values in the natural state condition are much higher than that in the air-dried condition. The results highlight that, the sandy soil layer and the detritus layer are easily to disintegrate compare with the surface layer and the red soil layer, and in the case of low soil water content, the soil in any layer of collapsing gully is easy to disintegrate. The regression equation shows a very significant and positive relationship between the *Kc* values and the < 0.002 mm particles contents and the SOM (soil organic matter) (*p*<0.01), and negative relationship between the *Kc* values and the contents of other soil particle size. The results revealed that the repulsive force produced by compressed air in the soil exceeds the suction between the soil particles is the predominant factor to soil disintegrate rates in the air-dried state condition. Whereas the soil contained a certain amount of water can reduce the degree of disintegration. The results also indicated that the more contents of the cementation agents (like clay and SOM) in the soil of the different layers of collapsing gully, the higher *Kc* values (it means the more difficult to disintegrate).

## Introduction

**Fig 1:**
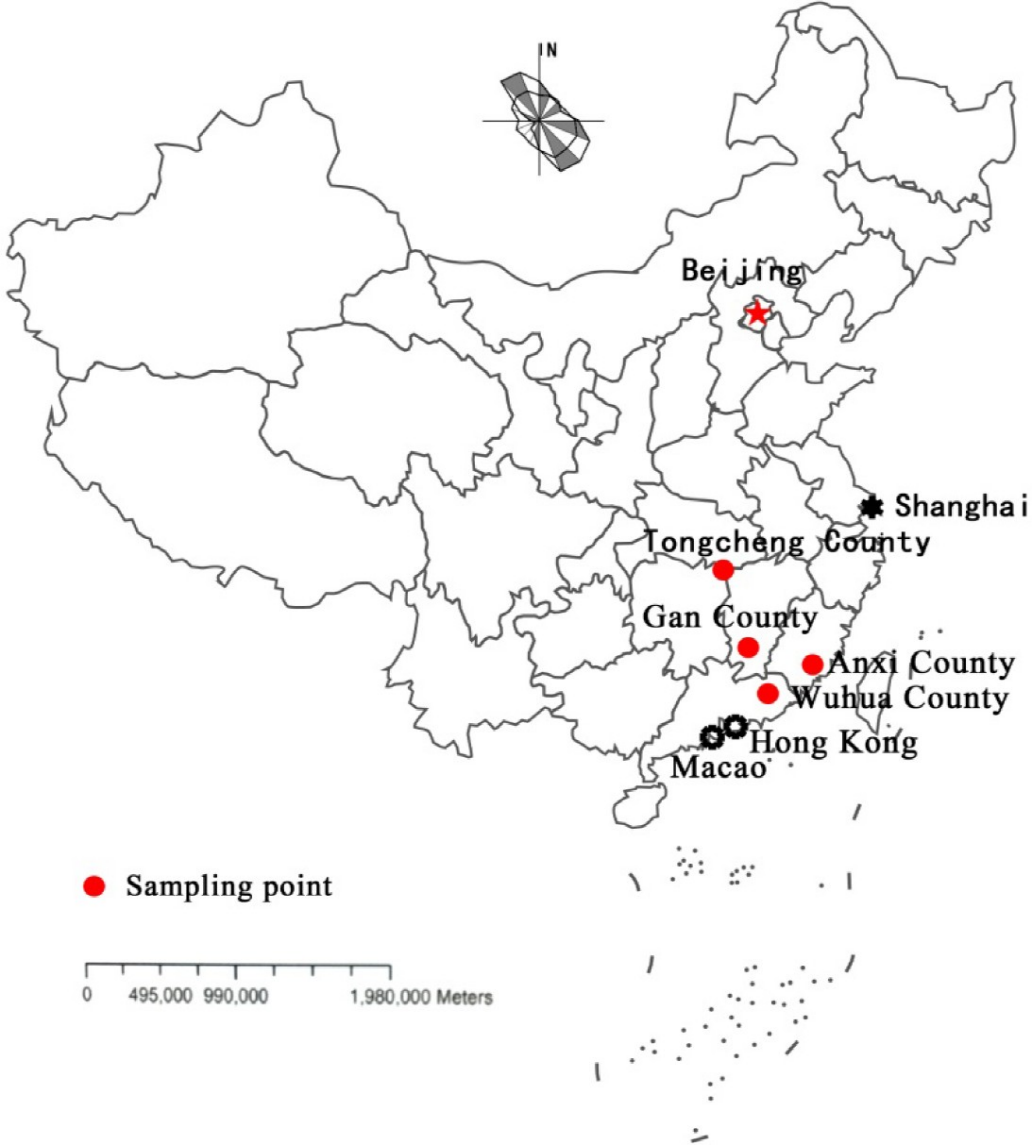
The sketch map of sampling sites. The sampling sites of Tongcheng county, Gan county, Anxi county and Wuhua county were represented by red dots on the China map. The representative cities, like Beijing, Shanghai, Hong Kong and Macao, were marked to reflect the relative positions of the sampling sites.

**Fig 2:**
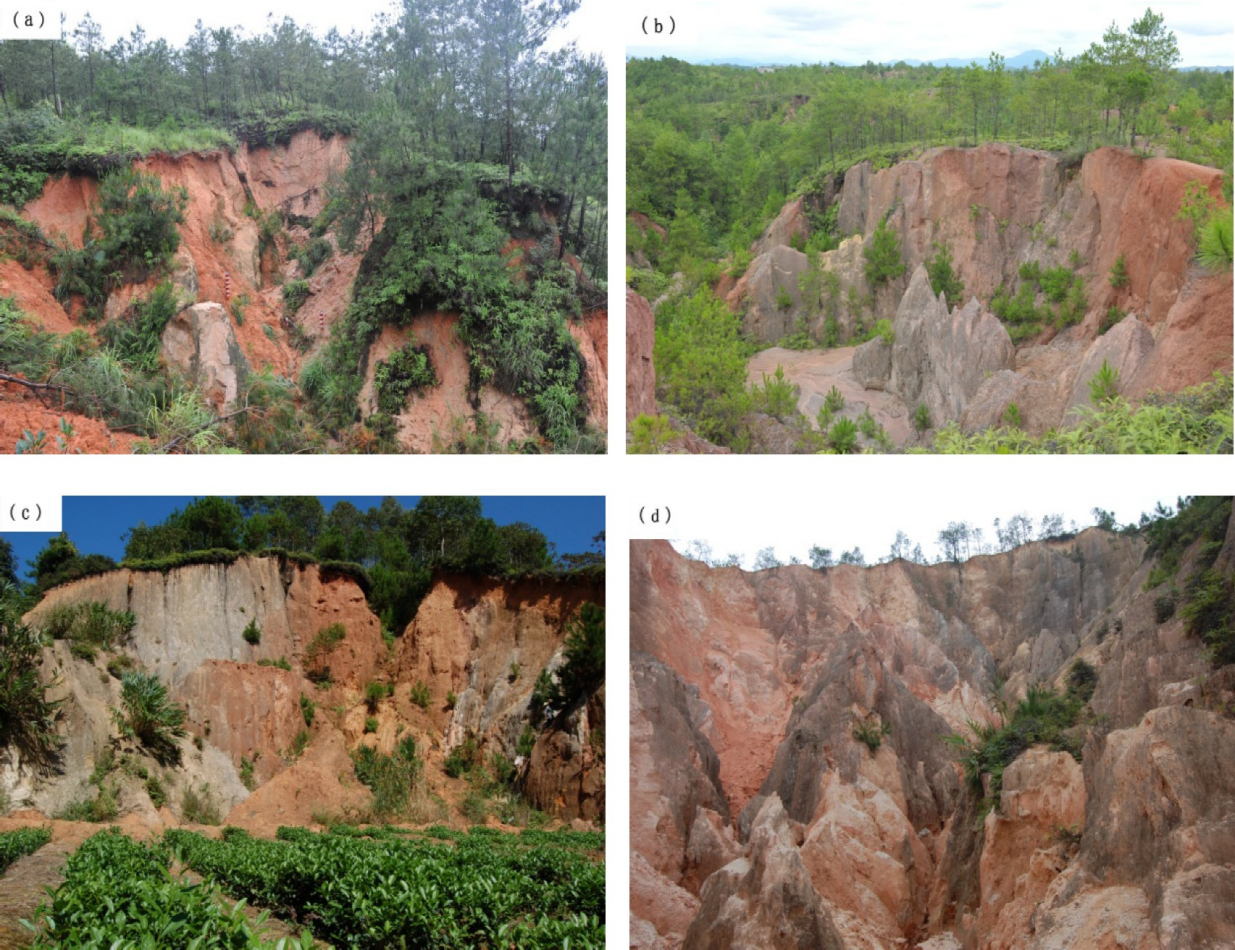
The typical collapse gully in the hilly granitic region (a: Tongcheng County; b: Gan County; c: Anxi County; d: Wuhua County) (photo: Dong Xia). The lowercase letters in the upper left corner of each Figure represented four collapsing gullies in the four counties, (a) Tongcheng County; (b) Gan County; (c) Anxi County; (d) Wuhua County.

The collapsing gully erosion is the main important and specific soil erosion type in the red soil region of tropical and subtropical South China [1–9] (Fig 1). Especially, on the granitic weathered crust, the collapsing gully is most extensive [5, 6, 8–13]. The sediment and the damage caused by collapsing gully is far greater than that of surface erosion and gully erosion, which destroys land resources and ecological environment and reduces the quality of farmland [13–16]. According to the obvious differences in the degree of weathering, soil color, soil structure and soil particle size distribution characteristics, the granitic weathering crust (collapsing gully wall) (Fig 2) can be subdivided into five profiles: surface layer, red soil layer, sandy soil layer, detritus layer and bedrock, from top to bottom [11, 17–21]. The soil disintegration property is one of the major erosion characteristics [6, 11, 22]. So, to understand the soil disintegration property of the granite weathering crust (that is, the collapsing gully wall, Fig 2) will be the necessary condition for finding out the mechanism of the forming process and development of the collapsing gully.

The soil disintegration refers to the phenomenon that the soil is dispersed after being immersed in water. With the infiltration of water, the cementation between the soil particles is weakened, and the soil structure become unstable, therefore soil disintegration occurs [23]. At present, a series of studies have been carried out on the disintegration characteristics of granite weathering soil, and the causes of soil disintegration have been analyzed [24–29]. Research demonstrates that the disintegrating process is an irreversible physical process [30]. The soil disintegration is one of the important characteristics of some special soil, which is affected by many factors [24, 31–33]. A number of researchers studied the influential factors of soil disintegration and found that soil organic matter contents (SOM) [25, 34–35], soil cementing substance contents (such as Ca^2+^, Fe^3+^, and Al^3+^, oxides and hydroxides of Fe and Al) [36–38], soil moisture [24, 38–39], soil texture (especially the clay content) [34, 36], soil crack [28], and soil porosity [26, 37] played important roles in the soil structure stability, and then affected the soil disintegration. Experiments conducted by Lado et al. [40] showed that aggregate stability increased with an increase in clay content, due to the cementing effect of the clay in the soil. Gamble [41] indicated that the change of the soil water content was the causation of soil disintegration, and meanwhile the SOM content could affect the disintegration characteristics. Liu [29] found that the dissolved of the stabilizing agents in soil would result decrease in bound strength between particles, and soil disintegrate. Zhang et al. [24] also reported that the soil disintegration rate significantly correlated with soil moisture content, and it decreased with an increase in initial soil moisture content. Lan et al reported that the gas extrusion theory can be used to explain the disintegration mechanism [23].

The soil disintegration rate is one of the evaluation indexes of soil erosion, which can be used to reveal some of the causes of the collapsing gully erosion [40, 42]. In recent years, some important research results have been carried out on the disintegration properties of granite weathering soil or the collapsing gully wall. Based on the physicochemical properties and microstructure of the granite weathered soil, Li described the development mechanism of collapsing gully and analyzed its disintegration process [43]. Although several studies have focused on the soil structure softening damage [26], disintegrating process [23,27,30] and disintegrating rate [27,44] of the granite residual soil, few researches that investigate the variations of soil disintegration features associated with soil layers in the hilly granitic region of southern China have been published in recent years. There is a need for quantitative information on the soil disintegration characteristics within different weathering profiles and its relationships with soil particle size distribution and soil properties in order to understand the mechanism of the forming process and development of the collapsing gully.

In this paper, the relationships between soil anti-disintegration index (*Kc*) and particle size distribution and SOM were analyzed based on the study of *Kc* in soils within the different soil layers (weathering profiles) of the four collapsing gullies (Figs 1 and 2). The objectives of this study are: 1) to evaluate the similarities and differences of the *Kc* of different soil layers of the four collapsing gullies located in different latitude; 2) to investigate the relationships between *Kc* and particle size distribution and SOM; 3) to explore the disintegration mechanism of different soil layers of collapsing gully under different soil moisture conditions.

## Materials and methods

### Collapsing gully sampling site selection

Four collapsing gullies in the four counties located in different latitude in the hilly granitic region, South China were selected according to the climate and geography conditions (Figs 1 and 2) [11]. Soils were sampled from the different weathering profiles of the four collapsing gullies in the four counties (Tongcheng County, Gan County, Anxi County and Wuhua County) (Figs 1 and 2). In this study, the counties’ names were used to name the four collapsing gullies respectively. In the hilly granitic region in South China, it has a typical subtropics monsoon climate, and from north to south, it is with mean annual temperature from 17°C to 22°C and an average annual precipitation from 1350 mm to1600 mm with high variability (the 75%-85% of the rain falls during March to September). According to statistics, the granite of the soil sampling site in the GX was formed in the Early Caledonian and Yanshanian, and the granites of the other three counties were formed in Yanshanian. In these regions, the granites are mostly coarse-grained granite and porphyritic granite, and mainly consist of feldspar and quartz [45–49]. So, there is no significant difference of the parent material of the granite. The other geological and geomorphological conditions, environmental and climate description of the study area please see the literature by Xia et al. [11] and Deng et al. [16].

### Soil sampling

The details of the soil sampling description of the four collapsing gullies can be see the literature by Xia et al. [11] and Deng et al. [16]. No specific permissions were required for the study area, for there were not a national park or other protected area of land, sea or wildlife. And the field studies did not involve endangered or protected species. The more than one hundred small soil blocks (diameter 3 to 5 cm) that obtained at each soil layer under one moisture content condition should meet the experiment repetition needs. In order to reduce the water content differences between the four collapsing gullies, the soil samples were obtained during a week without raining. The detailed descriptions of different soil layers and average moisture content are given in Table 1 and Table 2.

**Table 1:** Description of different soil layers of the collapsing gullies.

Soil layer	Tongcheng County		Gan County		Anxi County		Wuhua County	
	Soil layer code	Sampling height (m)	Soil layer code	Sampling height (m)	Soil layer code	Sampling height (m)	Soil layer code	Sampling height (m)
Surface soil layer	TC1	0–0.3	GX1	0–0.3	AX1	0–0.3	WH1	0–0.3
Red soil layer	TC2	0.8	GX2	0.8	AX2	0.8	WH2	1
	TC3	2	GX3	1.8	AX3	2		
							WH3	2.5
	TC4	4	GX4	4	AX4	4		
Sandy soil layer	TC5	7	GX5	7.5	AX5	8	WH4	5
	TC6	9	GX6	9	AX6	10	WH5	9
Detritus layer			GX7	11	AX7	12	WH6	11
			GX8	13.5	AX8	15	WH7	14
							WH8	17

**Table 2:** Description of average moisture content (kg kg^-1^) of different soil layers with two different moisture conditions.

Soil layer code	air-dried condition	natural state condition	Soil layer code	air-dried condition	natural state condition	Soil layer code	air-dried condition	natural state condition	Soil layer code	air-dried condition	natural state condition
TC1	0.085	0.240	GX1	0.054	0.206	AX1	0.039	0.184	WH1	0.040	0.161
TC2	0.046	0.193	GX2	0.041	0.189	AX2	0.044	0.256	WH2	0.033	0.165
TC3	0.087	0.248	GX3	0.029	0.208	AX3	0.038	0.198	WH3	0.040	0.151
TC4	0.078	0.204	GX4	0.045	0.205	AX4	0.030	0.247	WH4	0.037	0.171
TC5	0.054	0.202	GX5	0.037	0.246	AX5	0.034	0.173	WH5	0.034	0.145
TC6	0.070	0.223	GX6	0.018	0.221	AX6	0.052	0.245	WH6	0.043	0.173
			GX7	0.041	0.203	AX7	0.032	0.207	WH7	0.042	0.188
			GX8	0.035	0.244	AX8	0.039	0.214	WH8	0.047	0.190

### Soil analysis and formula

The soil particle size distribution was determined using the pipette method [50]. The submerging test was used for determining the anti-disintegration ability of soil blocks with two different moisture conditions (the air-dried condition and the natural state condition). This method required the soil blocks to be immersed in water. Fifty small soil blocks (diameter 3 to 5 cm) from different weathering profiles of the four collapsing gullies were randomly selected in the two moisture conditions respectively and placed uniformly on a screen with aperture mesh of 2 mm, then lowered the screen gradually into water and recorded the time to count the number of the disintegration within a certain time [51]. The anti-disintegration index (*Kc*) of soil is calculated according to the following formula [51–52]:

$$K_{c}=(a_{1}\times 5+a_{2}\times 15+a_{3}\times 25+\cdots +a_{10}\times 95+a_{\infty }\times 100)/50$$ (1)

The total immersion time is 10 minutes. The *a*_1_, *a*_2_, *a*_3_ … *a*_10_ express the accumulative number of complete disintegration small soil blocks at the immersion times of 1 min, 2 min, 3 min…10 min. *a*_∞_ is the number of small soil blocks which remain not totally disintegrated after an immersion time of 10 min. At the beginning of the first 1 minute, the water stability of the small soil block is 0. At the end of the tenth 1 minute, the water stability of the small soil block is 100. Then the water stability of the total immersion time of 10 minutes can be represent as 0, 10, 20, 30…100. The disintegration coefficient is an index to represent the anti- disintegration ability of small soil block in a period of time (1 minute). So we calculate the disintegration coefficient of the first 1 minute as 5 = (0+10)/2. If the small soil block disintegrates during the immersion time of 1–2 min, its coefficient is defined as 15 (= [10+20]/2). If the small soil block never disintegrates during the total immersion time of 10 min, its coefficient is defined as 100 [51–52]. The average value of triplicate measurements was obtained to compute *Kc* of soil. The increase in *Kc* value indicates that the more difficult to disintegrate (that is, the lower the disintegrate rate).

### Statistical analysis

All statistical analyses were carried out using the SPSS 16.0 statistical software package for Windows (SPSS, Inc., Chicago, IL, USA). Regression analysis was used to analyze the relationship between anti-disintegration index (*Kc*) and the contents of soil particles and soil organic matter. One-way analysis of variance (ANOVA) was used to test the differences of soil properties among soil layers. Differences at the *p*<0.05 level were considered significant. And significant differences were compared using the Fisher’s least significant difference test (LSD) for each soil variable.

## Results

### Soil particle size distribution

As described in detail previously [11], there are noticeable differences in soil particle size distributions among the different soil layers of the four collapsing gullies. The predominant soil particle size is found to be clay (< 0.002 mm) in the surface soil layer and the red soil layer, and it decline with the increasing depth. From surface layer to detritus layer, the sand particles (2.0–1.0 mm) in the TC and AX collapsing gully decrease firstly and then increase, but it roughly shows increase tend in the GX and WH collapsing gully. The contents of 1.0–0.25 mm particle in the TC, AX and WH show the falling tendency firstly and then increase from surface layer to detritus layer, but increases in the GX collapsing gully. The contents of 0.25–0.05 mm particle in the TC and GX increase firstly and then decrease from surface layer to detritus layer, but show the inversed order in the AX and WH. The 0.05–0.002 mm particle presents a fluctuated variation in the different soil layer of the four collapsing gullies. The detailed data please see the literature by Xia et al. [11].

### Soil anti-disintegration index (*Kc*)

**Fig 3:**
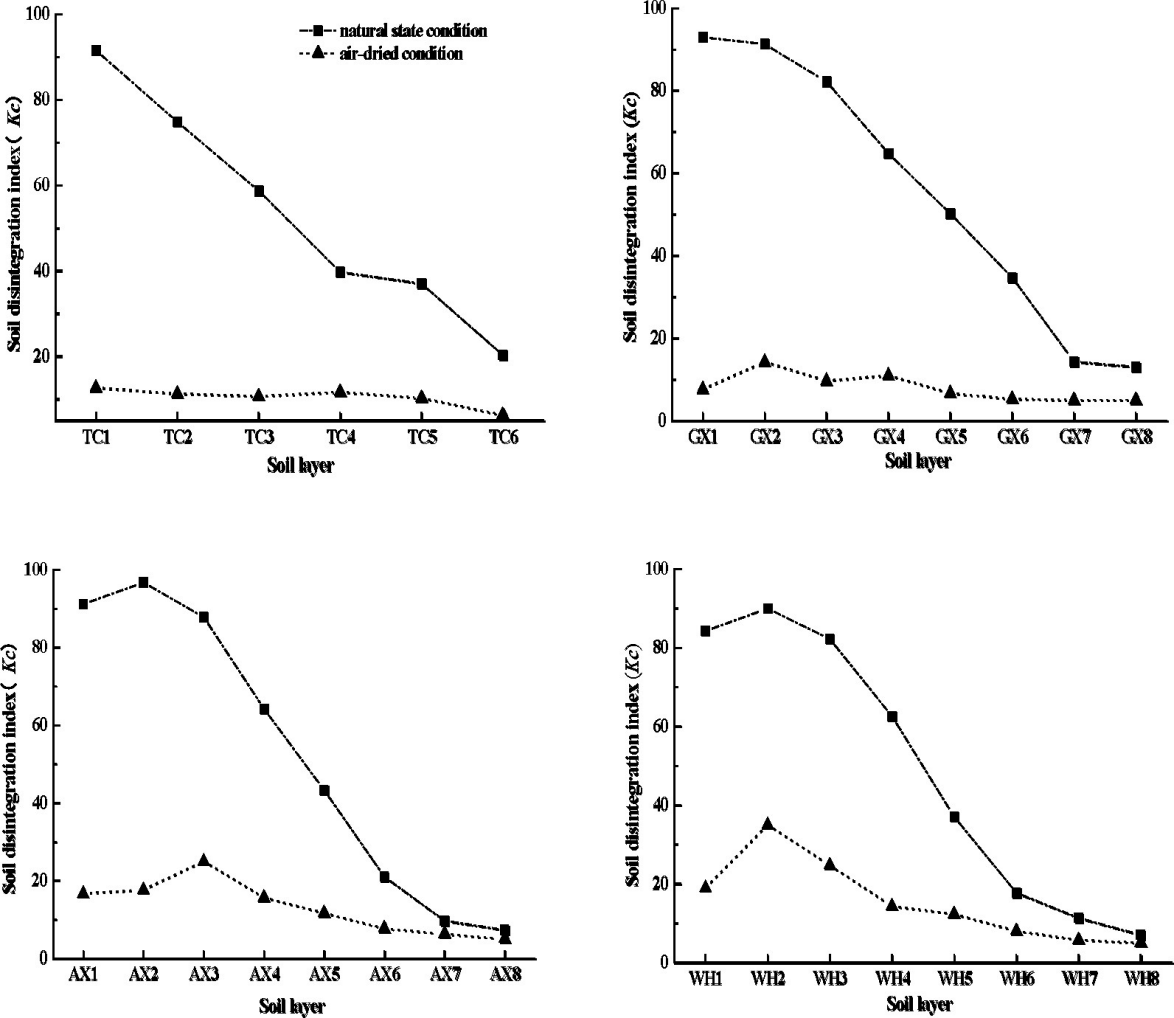
Variations in *Kc* of the different soil layers of the TC, GX, AX and WH collapsing gullies with two different moisture conditions. According to the *Kc* values change curves of the different soil layers of the TC, GX, AX and WH collapsing gullies with two different moisture conditions, the *Kc* values reduce significantly from the surface layer to the detritus layer. The *Kc* values in the air-dried condition are much lower than that in the natural state condition.

The variations in *Kc* values of the different soil layers of the TC, GX, AX and WH collapsing gullies with two different moisture conditions are showed in the Fig 3. The *Kc* values reduce significantly from the surface layer to the detritus layer (from 91.5 to 20.3, 91.2 to 7.3, 93 to 13, 84.3 to 7, respectively, in the natural state condition; from12.7 to 6.3, 16.7 to 5, 7.7 to 5, 19 to 5, respectively, in the air-dried condition), and these reveal that the detritus layer is the most prone to disintegration. The *Kc* values in the air-dried condition are lower than that in the natural state condition. Under the natural state condition, the *Kc* appear the peak value in the AX2 layer (96.7) and WH2 layer (90), respectively, and meanwhile the peak value of *Kc* appear in the GX2 layer (14.3), AX3 layer (25) and WH2 layer (35), respectively, with the air-dried condition (Fig 3). In the surface layer and the red soil layer, the *Kc* values in the natural state condition are much higher than that in the air-dried condition. And no significant difference about the *Kc* values is found between the two moisture conditions in the detritus layer. These results highlight that, in the case of low soil water content, the soil in any layer of collapsing gully is easy to disintegrate. Meanwhile, with natural state condition, there are high *Kc* values in the surface layer and the red soil layer, and it indicates that soil in these two layers is difficult to disintegrate. Our result is in agreement with the finding of Zhou and Li [28], who found that the sandy soil layer and the detritus layer had higher disintegration rates compare with the surface layer and the red soil layer.

### Correlations between the *Kc* and the soil particle-size distribution and SOM

**Fig 4:**
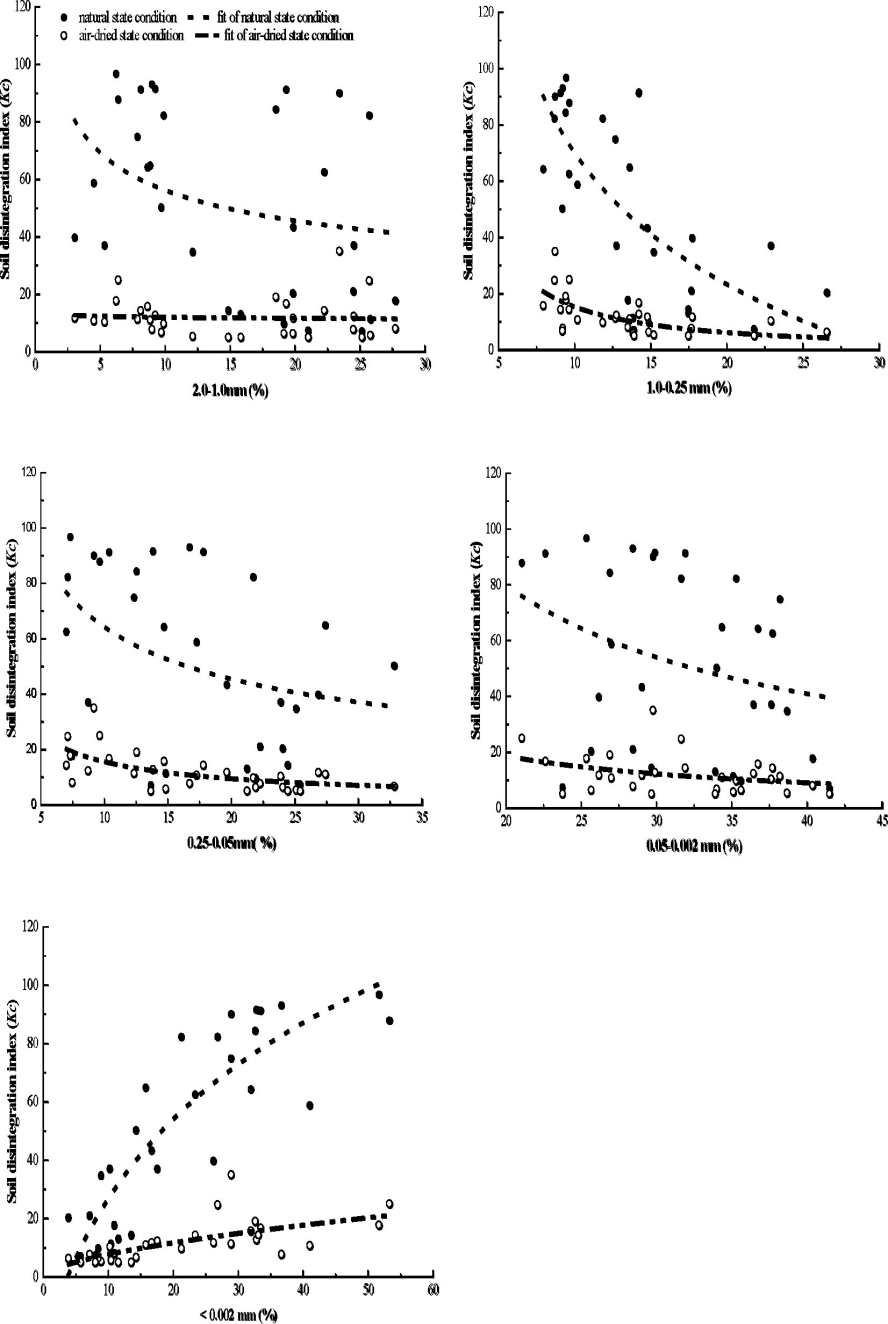
Correlations between *Kc* and the contents of the different soil particle-size distribution under the two different moisture conditions. Non-linear regression analyses results indicate that the *Kc* values had negative correlation with the 2.1–1.0 mm, 1.0–0.25 mm, 0.25–0.05 mm and 0.05–0.002 mm contents under the two different moisture conditions. But a very significant and positive relationship exists between the *Kc* values and the contents of < 0.002 mm.

**Fig 5:**
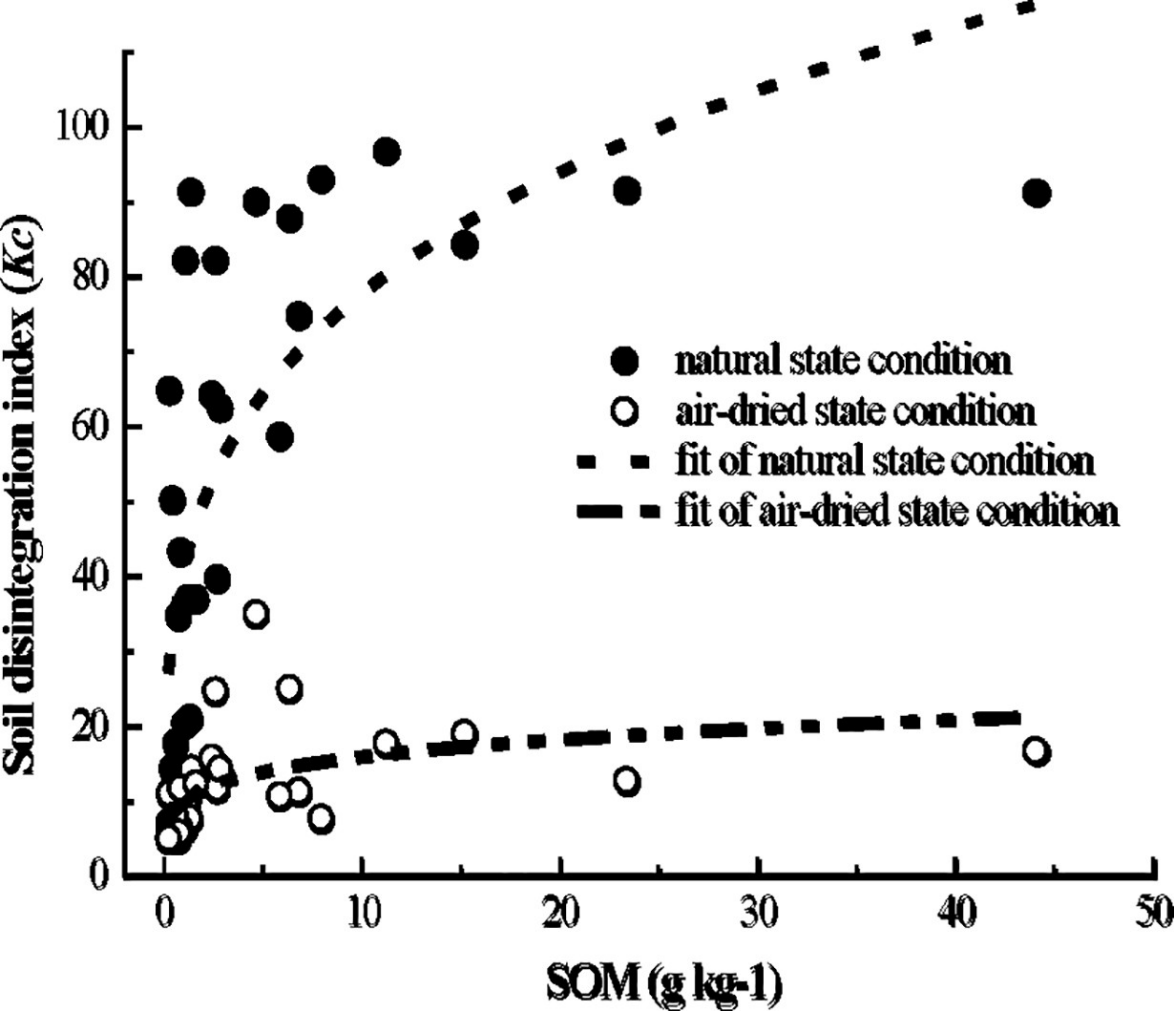
Correlations between *Kc* and the SOM under the two different moisture conditions. A highly significant and positive relationship exists between the *Kc* values and the SOM under the two different moisture conditions.

The early study of this experimental area [11] showed that the soil organic matter content was extremely low in the sandy soil layer and the detritus layer. The result is consistent with the studies about the distribution of soil organic matter in the different soil layers of the collapsing gullies in other experimental regions, which found that the soil organic matter contents sharply declined from the surface layer to the detritus layer [20, 28, 42]. Non-linear regression analyses were performed to determine the strength of relationships between *Kc* values and the contents of the different soil particle-size distribution under the two different moisture conditions (Fig 4), as well as the relationships between *Kc* values and the SOM (Fig 5), and they meet the logarithmic relationship or a power function relationship (Figs 4 and 5, Table 3). The results of regression analyses indicated that the *Kc* values had a weak negative correlation with the 2.0–1.0 mm and 0.05–0.002 mm contents under the two different moisture conditions (Table 3). Under the natural state condition, the results demonstrated a strong negative correlation with the contents of 1.0–0.25 mm (R^2^ = 0.4435, *p* < 0.01) and a remarkable negative correlation with the contents of 0.25–0.05 mm (R^2^ = 0.1419, *p* < 0.05) (Fig 4 and Table 3). Meanwhile a highly significant and negative relationship between the *Kc* values and the contents of 1.0–0.25 mm and 0.25–0.05 mm were found under the air-dried state condition (*p* < 0.01, R^2^ = 0. 3754 & 0.3500, respectively). The results of regression analyses also indicated that the contents of the < 0.002 mm and the SOM played an important role in the soil disintegration. Under the two different moisture conditions, regression equation showed a very significant and positive relationship between the *Kc* values and the < 0.002 mm and the SOM (*p* < 0.01). The results show that the higher the contents of the < 0.002 mm and the SOM, the more difficult the soil is to disintegrate, which are consistent with recent research findings [53–56].

**Table 3:** Correlations between soil anti-disintegration index (*Kc*) and soil particle-size distribution and SOM with two different moisture conditions.

Soil particle-size distribution(mm)	Natural state condition		Air-dried state condition	
	Regression equation	R^2^	Regression equation	R^2^
2.0–1.0	y = 112.55x^-0.301^	0.091	y = 13.27x^-0.042^	0.033
1.0–0.25	y = 155.78–47.77ln(x-3.99)	0.4435**	y = 309.36x^-1.305^	0.3754**
0.25–0.05	y = 202.47x^-0.499^	0.1419*	y = 81.37x^-0.717^	0.3500**
0.05–0.002	y = 1431.46x^-0.963^	0.0613	y = 418.49x^-1.034^	0.0763
<0.002	y = -146.71 +60.55ln(x+7.57)	0.7497**	y = 1.965x^0.5971^	0.4149**
SOM	y = 41.63x^0.272^	0.4907**	y = 10.22x^0.194^	0.2446**

*, *P* < 0.05

**, *P* < 0.01

## Discussion

### Effects of SOM on soil disintegration

SOM is an important factor affecting the formation of soil structure [57–59], as well as in the stabilization of soil aggregates [34, 60–65]. Generally, the level of aggregation and the stability of aggregates increase with increasing organic matter content [66–67]. Previous studies have suggested that the high organic content in soil is beneficial to the increase of soil anti-erodibility and decrease the disintegration of soil particles, which is of great significance to reduce erosion of soil [59, 63–64, 68].

Liu et al. [42] and Zhou and Li [28] indicated that the contents of SOM is closely related to the disintegration property of soil in the different soil layers, and the lower SOM contents in soil layer, the higher disintegration rate appeared. As well as, the results of Deng et al. [14] research showed that SOM can promote the formation of fine particles, subsequently the stability of soil aggregate enhanced. Previous studies revealed that SOM contents are extremely lower in the sandy soil layer and the detritus layer than that in the surface soil layer and the red soil layer [11], which is not conducive to the formation and stabilization of soil aggregate, and then cause high soil disintegration rate and low soil anti-erodibility [20]. In this study, compare with the surface soil layer and the red soil layer which have high SOM contents, the soil in the sandy soil layer and the detritus layer are more easily to disintegrate under the two different moisture conditions (Fig 5). The reason for this finding may be that SOM increase the stabilization and decrease the disintegrating speed of soil particles (Fig 5).

In soils, the inorganic stabilizing agents include mainly clays, polyvalent metal cations such as Ca^2+^, Fe^3+^, and Al^3+^, oxides and hydroxides of Fe and Al, calcium and magnesium carbonates and gypsum [38]. Researches indicate that free Fe and Al oxides play a role in soil cementation and are conducive to the stability of soil microstructure, meanwhile soil aggregates can be dispersed by Na^+^ and K^+^ [28, 36–38]. Other studies also show that the cementing effect of free Fe and Al oxides is important in soils with low organic matter content [37, 69]. Micro aggregates exhibit high stability when they are formed by binding of multivalent cations (Ca^2+^, Fe^2+^ and Al^3+^) and humus. Conversely, if micro aggregates are bound by the Na^+^ cations, or if there is a dispersion of clay particles, the soil structure becomes unstable [37–38, 70]. The studies of Zhang et al. [21] and Zhou and Li [28] indicate that the contents of free Fe and Al oxides in the surface soil layer and the red soil layer are higher than that in the sandy soil layer and the detritus layer, and the contents of Na^+^ and K^+^ cations have the opposite trend with free Fe and Al oxides. These results can also explain why the sandy soil layer and the detritus layer are more easily to disintegrate than the surface soil layer and the red soil layer to some extent.

### Effects of particle size distribution on soil disintegration

The hilly granitic region of tropical and subtropical South China is known for its thick granite weathering crust, with the minimum soil thickness being more than 20 m [23]. From the surface soil layer to bottom, the degree of weathering granite weathering crust decline with soil depth. In this sequence, the grain size becomes coarser, and the content of clay and organic matter decreases [5, 11, 18]. Our previous results show that the finer soil particles declined and the coarse soil particles increased with the declining of weathering degree (from the surface layer to the detritus layer) [11]. These results confirmed that the laterization and weathering degree of parent material significantly affected the rock-soil characteristics of granite weathering crust [11, 18, 28].

Recent studies show that the higher the coarse grain content in the granite weathered soil, the more likely the soil will collapse, and the higher the fine particles content, especially the higher the clay content, the stronger the soil water stability will be. With the decrease of clay content, the disintegration rate of soil is accelerated [23, 29–30, 55–56, 71].

Considerable researches confirm that the clay content is beneficial to soil structure stability and soil aggregates water stability [12, 40, 72–73], which are also found in our research (Fig 4). Research of Deng et al. [12] suggested that the soil water stability and the soil structure stability decline from top layer to bottom of the collapsing gully wall. Clay particles are enriched in secondary minerals that generally possess a higher soil particle density than quartz material, which for many soils dominate the silt and especially the sand fraction [73]. Rühlmann et al. [72] found a similar trend with soil particle density decreasing for soils with increasing content of sand. Lado et al. [40] also demonstrate that the soil stability was increased in the soil have high clay content, because the clay enhances the cementation in the soil and reduce the disintegrating rate of soil. Our result in this paper is in agreement with the finding of Lado et al. [40].

The cohesive process between soil particles is very weak for the soil layer in the granite weathered crust (collapsing gully wall), which has the high sand content. And in the process of soil drying or saturating, the force between the soil particles further weakens and leads to soil instability and is prone to collapse in the case of touching water. Meanwhile, the more content of coarse particles, the easier to form large soil porosity which is conducive to water entry, thus causing the soil disintegration [29]. Given the above reasons, soil particle size distribution can affect soil disintegration rate of the different soil layer. Our results indicate that the sandy soil layer and the detritus layer of collapsing gully have the high coarse particles and low clay content, so both these two layers are easily to disintegrate under the two different moisture conditions (Fig 4).

### Effects of soil moisture condition on soil disintegration

The soil moisture content has important influence on the stability of soil aggregates and soil structure [28, 40, 74–75]. A recent study found that soil slaking occurs when the aggregate is exposed to stresses produced by differential swelling, entrapped air explosion, rapid release of heat during wetting, and the mechanical action of moving water [40]. The soil water correlates to the thickness of hydrated films surrounding the soil particles, namely the diffusion layer, which has effect on the suction among the soil particles [75]. This fact clearly indicates that the initial condition especially water content in unsaturated state can influence the behavior of unsaturated soils [76]. Meanwhile the increase of initial water content will decrease the effective void ratio, thereby narrowing the seepage space and weakening the intrusion effects [74–75]. Kim and Kim [76] found that the water content cannot influence the effective cohesion significantly at high suction levels (that is, the water content decreases). Xu et al. [75] also indicated that, in certain water content, the increase of initial soil moisture content can obviously strengthen the resistance ability of soil aggregates and reduce the degree of disintegration of aggregates.

This phenomenon can be attributed to a decrease in slaking forces when the soil contains a certain amount of water. The volume of air entrapped decreases with increasing soil antecedent water content, which result in lower compression forces acting on the aggregates during immersed under water and fast wetting [77]. Some studies suggested that fast wetting of the air-dried soil caused considerably more slaking compared with the moist soil [40, 74–75, 77]. Shi et al. [78] also indicated that soil slaking appeared to be the dominant breakdown mechanism in initially dry soil and was particularly strong at the higher rainfall intensity. This can be explained that drying of the soil will increase the mechanical resistance to detachment and aggregate breakdown [77]. Meanwhile, unequal pressures caused by differential swelling during rewetting and the presence of entrapped air in pores also promote aggregate breakdown [79]. However, when the soil contains a certain amount of water, the initial soil water avoids entrapment of air and reduces spatial swelling differences, thereby increasing aggregate stability [77, 80].

Compare the *Kc* values of the same soil layer under the two different soil moisture conditions, it finds that the *Kc* values of the surface layer and the red soil layer in the natural state condition are much higher than that in the air-dried, but no significant difference about the *Kc* values are observed between the two different soil moisture conditions in the detritus layer (Fig 3). These results indicate that soil moisture significantly affects the soil disintegration characteristics of the surface layer and the red soil layer, but is not the dominant factor that affects the *Kc* values in the detritus layer between the two moisture conditions (the air-dried condition and the natural state condition). It also confirm that the same soil layer of the different collapsing gully has the similar soil disintegration characteristics, and in the case of low soil water content (the air-dried state condition), the soil in any layer of the four collapsing gullies is more easily to disintegrate compare with the natural state condition (Fig 3). These results are consistent with the results of Vermang et al. [77] and Bissonnais [80] that the initial soil water can increase soil aggregate stability and thereby decreasing the soil disintegrate. And these results are also in agreement with the finding of Zhou and Li [28] and Lado et al. [40] that the high coarse particles and low clay content is the dominant factor that affects the soil disintegration characteristics of the sandy soil layer, especially of the detritus layer. The overall trends of the *Kc* value of four different collapsing gullies are decreasing from the surface layer to the detritus soil layer under the two different moisture conditions. Thus the discussion of the relationship between the soil disintegration and the soil moisture of the same soil layer among two different moisture conditions can better reflect the influence of soil moisture on the *Kc* value.

### Analysis of disintegration characteristics of different soil layers of collapsing gully

**Fig 6:**
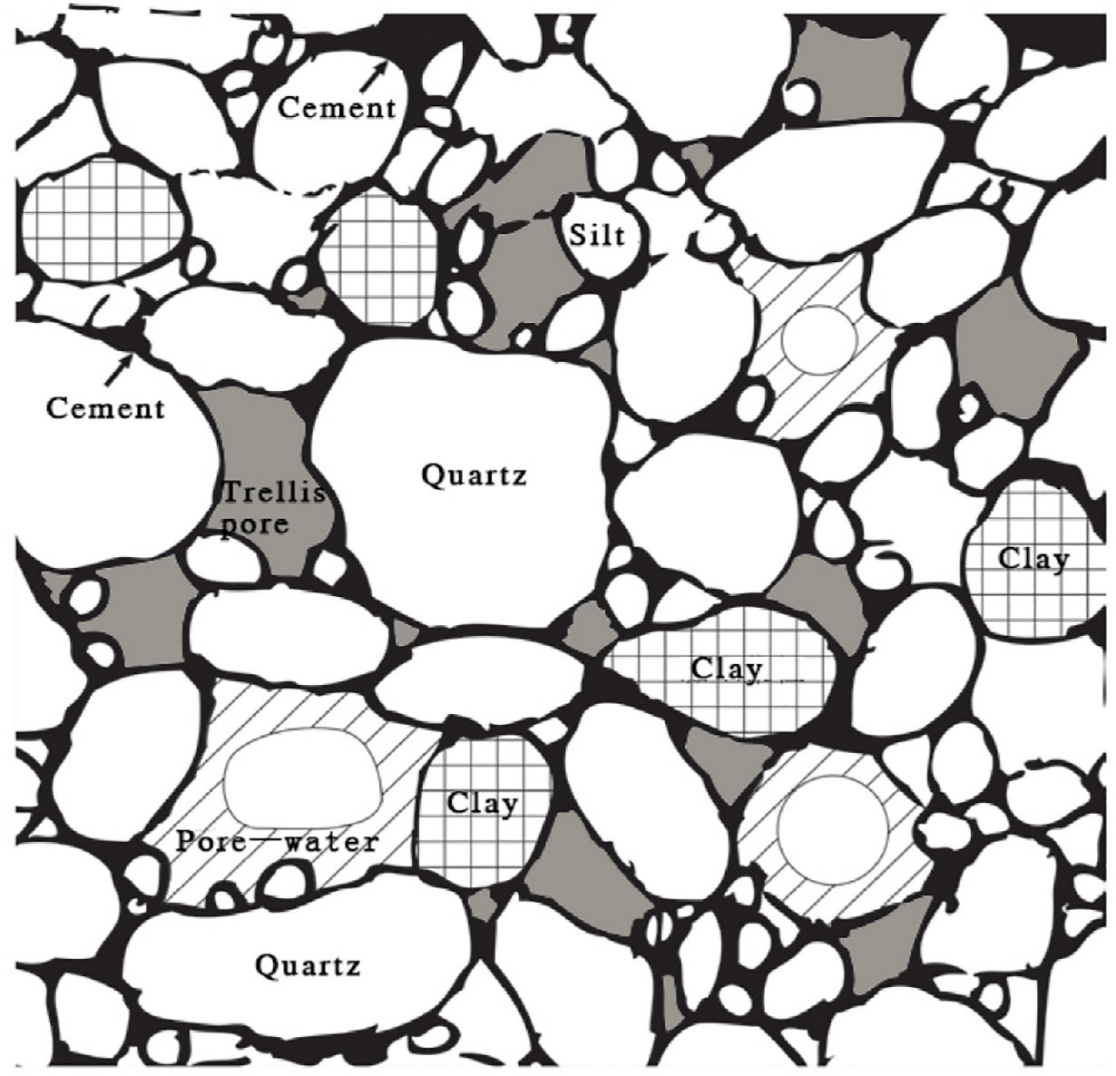
Soil structure of granite residual soils [23]. The stable quartz grains compose soil as skeleton material, combining with other cementing materials and fine particulate matter in various ways, forming a unique soil structure of granite weathering crust (collapsing gully wall).

Previous studies have suggested some explanations for the disintegration mechanism and influential factors of the collapsing gully soil as follow: a) the first is gas extrusion theory. Some researches indicated that a portion of the soil pores air was entrapped and compressed by the infiltrating water when the water entering the soil and infiltrating into the pores, thereby resulting an increasing in pressure of the pores air. Subsequently, soil disintegration occurs due to soil particles collapsed by entrapping air explosion and changing in soil structure (Fig 6). In generally, it is that the repulsive force produced by compressed air in the soil exceeds the suction between the soil particles [24–25, 80]. b) A portion of studies suggested that the essential reason for disintegration was softening damage. In the process of water absorbing, the inhomogeneity of soil particle size and pore size make the soil suction unbalance, and then lead to soil structure softening damage [26–27]. c) The stabilizing agents, such as Ca^2+^, Fe^3+^, and Al^3+^, oxides and hydroxides of Fe and Al, that play an important role in soil structure stability [26, 28–29]. In the meantime, due to the involvement of water, the water film between the soil particles are thickened, and then the suction between the soil particles can also be reduced, as a result, led to soil disintegrate [23, 81–82]. In addition, a new study also concluded that soil cracks also have an important influence on disintegration. Water can easily permeate into the soil through the cracks, therefore speeds up the contact between water and soil particles and then lead to soil disintegration [28].

In the soil layer of granite weathering crust, there are a certain amount of stable quartz grains, which compose soil as skeleton material, combining with other cementing materials and fine particulate matter in various ways, forming a unique soil structure of granite weathering crust (collapsing gully wall) [5–6, 22–23] (Fig 6). As described in detail previously, the coarse particles of the sandy soil layer and the detritus layer are higher than that in the surface layer and the red soil layer, but the finer particles show the inversed order [11]. Under the two different moisture conditions, the sandy soil layer and the detritus layer are easily to disintegrate compare with the surface layer and the red soil layer, whereas the *Kc* value of each soil layer in the air-dried condition was much lower than that in the natural state condition (Fig 3). The regression equations show a very significant and positive relationship between the *Kc* values and the contents of the < 0.002 mm and the SOM (*p* < 0.01) (Figs 3 and 4, Table 3). These results confirm that soil physic-chemical property, particle size distribution (especially the cementation agents, like soil organic matter, clay) and soil moisture contents are the dominant factors to influence the disintegration characteristics of the surface layer and the red soil layer of collapsing gully. And compared to soil physic-chemical property and particle size distribution, soil moisture content do not play a major role in affecting the disintegration characteristics of the sandy soil layer and the detritus layer.

Based on the results, the disintegration mechanism of different soil layers of collapsing gully is discussed.

1. Disintegration mechanism under different soil moisture conditions: In the air-dried condition, due to the evaporation of water, the water film thickness between the soil particles declines, thereby the cementation between soil particles decreases, meanwhile the cracks increase. Under this condition, the pores in the soil are mostly occupied by the air, and the internal pore suction is much larger than that of the soil which contains certain water (in the natural condition). When these air-dry soils are immersed in water, a portion of the soil pores air is entrapped and compressed by the infiltrating water, subsequently, the entrapped air increases the pressure and slaking forces. Overall, the repulsive force produced by compressed air in the soil exceeds the suction between the soil particles, so the resultant rapid release of energy and unequal pressures cause considerable soil disintegration. In this paper, our results confirm that the *Kc* values of each soil layer in the air-dried condition of collapsing gully are much lower than that in the natural state condition (Fig 3). It means that the soil of collapsing gully with lower water content is more likely to disintegrate once touching water or rainfall.
2. Disintegration mechanism under different soil properties: The coarse particles in the sandy soil layer and the detritus layer are higher than that in the surface layer and the red soil layer. In the sandy soil layer and the detritus layer, the cohesive force between the soil particles decline due to low cementation agents (such as free Fe and Al oxides, SOM and clay, etc.), which lead the sandy soil layer and the detritus layer to be unstable structure soil. When the soil of the sandy soil layer and the detritus layer touch water or rainfall, the hydrated films surrounding the soil particles, namely the diffusion layer, have a weakening effect on the suction among the soil particles, subsequently, the soil disintegrate. Conversely, the surface layer and the red soil layer have the high values of cementation agents (especially the free Fe and Al oxides, SOM and clay content), so their stability are higher compare with the sandy soil layer and the detritus layer (that is, the surface layer and the red soil layer are not easily to disintegrate). Our research confirms this point, the *Kc* values of the surface layer and the red soil layer are higher than that of the sandy soil layer and the detritus layer both under the two moisture conditions (Fig 3). The results reveal that the sandy soil layer and the detritus layer are much easily to disintegrate than the surface soil layer and the red soil layer once touching water or rainfall.

## Conclusion

1. In general, in the sandy soil layer and the detritus layer of collapsing gully, the coarse particles are higher than that in the surface layer and the red soil layer, but the finer particles show the inversed order.
2. Under the two different moisture conditions, the sandy soil layer and the detritus layer are easily to disintegrate compare with the surface layer and the red soil layer, whereas in the air-dried condition, the *Kc* values of each soil layer are much lower than that in the natural state condition. The regression equations show a very significant and positive relationship between the *Kc* values and the contents of the < 0.002 mm and the SOM (*p* < 0.01), and negative relationship between the *Kc* values and the contents of other soil particle size. The results of regression analyses indicate that the contents of the < 0.002 mm and the SOM play the important role in the soil disintegration of different soil layers of collapsing gully.
3. Based on the research under different soil moisture conditions and different soil properties, the disintegration mechanism of different soil layers of collapsing gully is discussed. In the air-dried state condition, when these air-dry soils are immersed in water, the repulsive force produced by compressed air in the soil exceeds the suction between the soil particles, and this is the predominant factor leading to soil disintegrate. Whereas the soil contains a certain amount of water can reduce the degree of disintegration. That means, in the lower water content, the soil of collapsing gully is more likely to disintegrate once touching water or rainfall. And under different soil properties, the contents of cementation agents, clay and the SOM play the important role in the soil disintegration. The results show that the more contents of the cementation agents in the soil of the different layers of collapsing gully, the higher *Kc* values (the more difficult to disintegrate).

## Supporting information

S1 Table Soil particle size distribution and soil anti-disintegration index of different weathering profiles of the four collapsing gullies. (XLSX)
